# The mysterious association between adiponectin and endometriosis

**DOI:** 10.3389/fphar.2024.1396616

**Published:** 2024-05-15

**Authors:** Yong-Qing Zhao, Yi-Fan Ren, Bing-Bing Li, Chao Wei, Bin Yu

**Affiliations:** College of Integrated Chinese and Western Medicine, Jining Medical University, Jining, Shandong Province, China

**Keywords:** adiponectin, endometriosis, apoptosis, angiogenesis, inflammation, fibrosis, metabolism, estrogen

## Abstract

Adiponectin is a pleiotropic cytokine predominantly derived from adipose tissue. In addition to its role in regulating energy metabolism, adiponectin may also be related to estrogen-dependent diseases, and many studies have confirmed its involvement in mediating diverse biological processes, including apoptosis, autophagy, inflammation, angiogenesis, and fibrosis, all of which are related to the pathogenesis of endometriosis. Although many researchers have reported low levels of adiponectin in patients with endometriosis and suggested that it may serve as a protective factor against the development of the disease. Therefore, the purpose of this review was to provide an up-to-date summary of the roles of adiponectin and its downstream cytokines and signaling pathways in the aforementioned biological processes. Further systematic studies on the molecular and cellular mechanisms of action of adiponectin may provide novel insights into the pathophysiology of endometriosis as well as potential therapeutic targets.

## 1 Introduction

Endometriosis (EMs), which is considered to be a benign form of gynecological cancer, is a common gynecological disease caused by the invasive growth and development of active endometrial tissue at sites outside of the uterus (Taylor et al., 2021). EMs is often accompanied by chronic pelvic or sexual pain, dysuria, infertility, dysmenorrhea, constipation, anxiety, and depression, among other symptoms, all of which can seriously affect the quality of life and the physical and mental health of patients (Bulun et al., 2019; Zondervan et al., 2020; Lamceva et al., 2023). In recent years, the incidence of EMs has markedly increased, affecting approximately 10%–15% of women of childbearing age globally, with a prevalence of up to 50% of women with infertility and 50%–80% of women with pelvic pain (Mehedintu et al., 2014; Zondervan et al., 2018; Taylor et al., 2021). The “retrograde menstruation theory,” which was first proposed by Sampson in 1927, is the classical doctrine describing the etiology of EMs (Sampson, 1927). While the incidence of retrograde menstruation is estimated to be approximately 90% in women of childbearing age, the much lower proportion of women affected by EMs suggests that other factors may be involved in its pathogenesis (Czyzyk et al., 2017). Inflammatory factors, immune dysregulation, angiogenesis, fluctuating hormone levels, genetic and epigenetic factors, environmental factors and other mechanisms, may contribute to the onset of the disease. In recent years, the study of EMs has become a hot spot of gynecological research, but its specific pathogenetic mechanisms are not yet fully understood.

Adiponectin is a pleiotropic cytokine that is predominantly secreted by adipose tissue and was initially considered to be an important insulin sensitizer and regulator of energy homeostasis (Wang and Scherer, 2016; Straub and Scherer, 2019). In recent years, an increasing number of studies have investigated the role of adiponectin in many diseases processes, and there is growing evidence of its involvement in the regulation of apoptosis, inflammation, angiogenesis, and fibrosis (Bråkenhielm et al., 2004; Fang and Judd, 2018), and adiponectin may be associated with estrogen-related diseases (Rizzo et al., 2020; Tsankof and Tziomalos, 2022), all of which contribute to the pathogenesis of EMs. Many studies have reported the presence of low adiponectin levels in those with EMs. The purpose of this review was to provide a detailed and up-to-date overview of the role of adiponectin in apoptosis, autophagy, inflammatory responses, angiogenesis, fibrosis, energy metabolism, and estrogen-related processes, and its potential correlation with the pathogenesis of EMs.

## 2 Obesity, adipose tissue, and EMs

The overall degree of obesity or the distribution of adipose tissue has been shown to be correlated with the development of EMs (Shah et al., 2013b; Backonja et al., 2016). For example, Goetz et al. demonstrated that the expression levels of four genes known to be associated with weight loss [cytochrome P450 2R1 (CYP2R1), fatty acid binding protein 4 (FABP4), mannose receptor C-Type I (MRC1), and Rho-associated coiled-coil containing protein kinase 2 (ROCK2)] were upregulated in the livers of mice in an animal model of EMS, whereas the expression levels of two genes associated with obesity [insulin-like growth factor binding protein 1 (IGFBP1) and monocyte to macrophage differentiation associated 2 (MMD2)] were downregulated; in addition, the presence of EMs was associated with a reduction in both the level of body fat and the degree of weight gain (Goetz et al., 2016). Another study reported a decrease in the proliferative ability of abdominal subcutaneous adipocytes in patients with EMs (Zolbin et al., 2019). The inflammatory response has also been shown to be intensified in the retroperitoneal adipose tissue adjacent to lesions in patients with pelvic EMs, and these changes were accompanied by a significant upregulation of the expression levels of angiogenic factors and inflammatory cytokines (Kubo et al., 2021). In addition, a reduction in the number of adipose stem cells and lipid dysfunction have been reported in a mouse model of EMs, which can affect the body mass index (BMI) by modulating adipocytes and lipid metabolism (Zolbin et al., 2019). There is a large body of evidence that suggests the risk of EMs is negatively correlated with the BMI (Vitonis et al., 2010; Backonja et al., 2016; Farland et al., 2017; Holdsworth-Carson and Rogers, 2018; Pantelis et al., 2021), with this negative correlation being intensified in women with infertility (Shah et al., 2013a).

**FIGURE 1:**
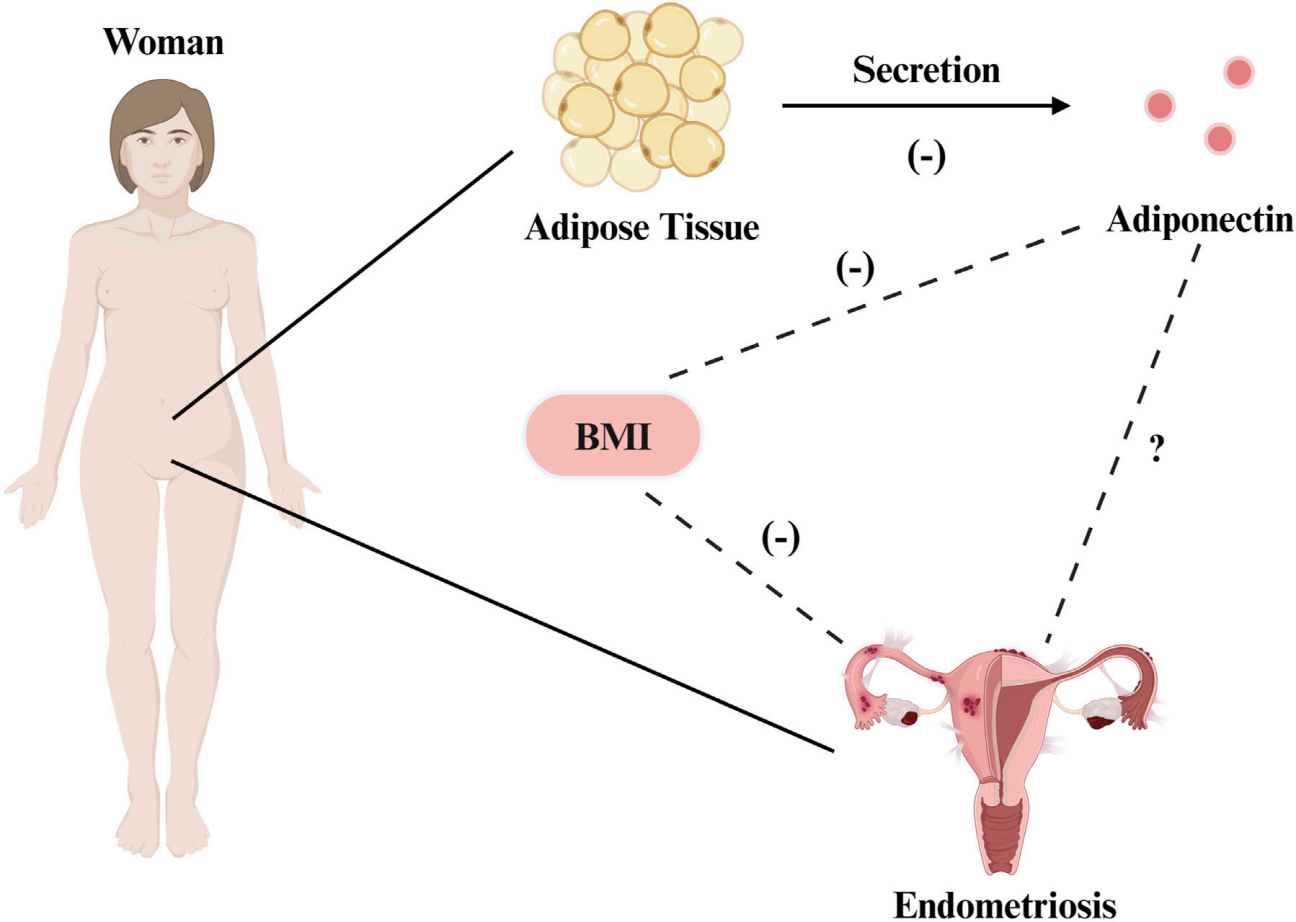
The correlation between EMs, obesity, and adipose tissue. There is a negative correlation between the risk of EMs and BMI. Adiponectin is produced by adipocytes, and its level is negatively corelated with BMl and fat accumulation. Evidence shows that adiponectin is underexpressed in endometriosis, and the two variables may exhibit a certain degree of correlation. Created with BioRender.com.

It is important to acknowledge, however, that some studies have reported that the BMI had no effect on endometrial gene expression in patients with EMs (Holdsworth-Carson et al., 2020), and the negative correlation between EMs and BMI remains unexplained, as both have been shown to be associated with hyperestrogenemia and systemic inflammation, and obesity does not prevent the occurrence of EMs (Holdsworth-Carson and Rogers, 2018; Pantelis et al., 2021). Some studies have suggested that in addition to being a risk factor for the disease (Tang et al., 2020), obesity may also exacerbate the condition in clinical populations (Holdsworth-Carson et al., 2018). Collectively, these findings suggest that multiple factors affect the correlations between EMs, obesity, and the presence of adipose tissue (Figure 1).

## 3 Adiponectin

### 3.1 Overview, structure, and oligomers

**FIGURE 2:**
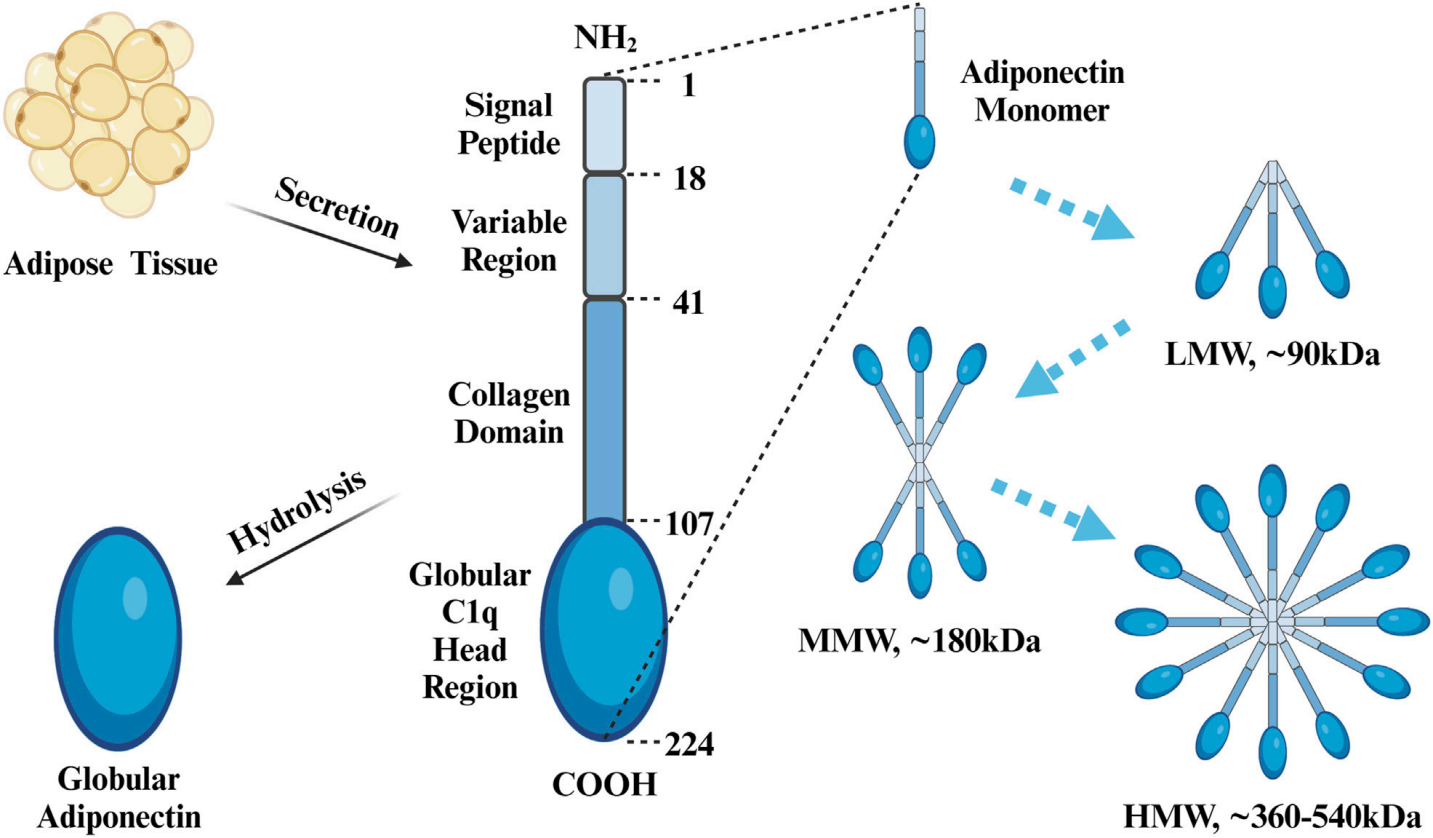
Protein Structure of APN. Adiponectin consists of an N-terminal signal peptide, variable region, collagen domain, and C-terminal globular c1q head region, with a total of 244 amino acids. Globular adiponectin is produced by proteolysis of full-length adiponectin. Three adiponectin monomers combine to form a trimer (LMW), two trimers combine to form a single hexamer (MMW), and four to six trimers combine to form a multimer (HMW). Created with BioRender.com.

Adipose tissue is a highly active endocrine organ that produces and expresses various factors such as leptin, adiponectin, and resistin, all of which participate in and coordinate a wide range of pathophysiological processes, including immune responses, inflammation, and energy metabolism (Kershaw and Flier, 2004). Adiponectin is an adipocytokine found in high abundance in peripheral blood, with a plasma concentration of 4–37 μg/mL, accounting for 0.01%–0.05% of total serum proteins, a concentration that is approximately a thousand times higher than that of other hormones, including insulin and leptin (Fang and Judd, 2018; Ye et al., 2020). Adiponectin has had many monikers over the years, including AdipoQ (Hu et al., 1996), apM1 (Maeda et al., 1996), Acrp30 (Scherer et al., 1995), and gelatin-binding protein 28 (GBP-28) (Díez and Iglesias, 2003). In humans, full-length adiponectin contains 244 amino acid residues and consists of an amino-terminal signaling peptide (amino acids 1–18), a variable region (amino acids 19–41), a collagen domain (amino acids 42–107), and a carboxy-terminal globular C1q head region (amino acids 108–244) (Achari and Jain, 2017). The isolated spherical C1q domain produced by protein hydrolysis, dubbed globular adiponectin, exhibits confirmed biological activity (Fruebis et al., 2001). As a monomer, adiponectin is difficult to detect in the blood (Waki et al., 2003), and three kinds of polymers can be found in the general circulation, including low-molecular weight (LMW) polymers (trimers of ∼90 kDa in size), medium-molecular weight (MMW) polymers (hexamers of ∼180 kDa), and high-molecular weight (HMW) polymers (chains of 12–18 monomers, with a size of ∼360–540 kDa) (Fang and Judd, 2018) (Figure 2). The LMW form can cross the filtration barrier within the kidneys; therefore, adiponectin can be detected in the urine of healthy individuals with normal renal function (Kim and Park, 2019). However, in the general circulation, HMW and MMW adiponectin are predominant, whereas concentrations of LMW adiponectin tend to be much lower (Parker-Duffen et al., 2013). Among them, HMW adiponectin is considered to be the main bioactive complex of the various isoforms (Achari and Jain, 2017). Globular adiponectin can also be detected in the plasma at low levels in the general circulation (Fruebis et al., 2001). Three adiponectin monomers combine via the C-terminal globular and collagen-like structural domains to form a highly ordered LMW trimer, which can further polymerize to form MMW hexamers and HMW compounds (Magkos and Sidossis, 2007). Circulating adiponectin levels are not constant, and serum concentrations have been shown to be higher during the daytime than at night (Gavrila et al., 2003; Scheer et al., 2010). In addition to these diurnal fluctuations, sex differences also influence adiponectin levels, with concentrations being higher in women than in men (Ohman-Hanson et al., 2016; Christen et al., 2018; Vučić Lovrenčić et al., 2020); this is particularly true in the case of HMW and MMW adiponectin, the levels of which are more than twice as high as in women (Peake et al., 2005). Higher circulating testosterone levels in males may explain the sex-specific difference in adiponectin concentrations after puberty (Handelsman et al., 2018), as testosterone lowers total adiponectin concentrations in serum (Nishizawa et al., 2002; Frederiksen et al., 2012; Yarrow et al., 2012). Although adiponectin is produced by adipocytes, significantly downregulated expression levels has been detected in the adipose tissue of fatty mice and overweight humans (Hu et al., 1996), with serum levels being negatively correlated with the BMI and fat accumulation (Brooks et al., 2007).

### 3.2 Adiponectin receptors and functions

Adiponectin was initially identified as an insulin-sensitive adipose factor related to the pathogenesis of metabolic syndrome; over time, however, many studies have confirmed its anti-inflammatory, anti-apoptotic, anti-fibrotic, and anti-angiogenic effects (Bråkenhielm et al., 2004; Fang and Judd, 2018). Adiponectin signal transduction mainly depends on receptor–ligand interactions, whereby adiponectin binds to its homologous receptor and initiates the activation of intracellular signaling cascades through the adenosine monophosphate-activated protein kinase (AMPK), peroxisome proliferator-activated receptor alpha (PPAR-α), phosphoinositide 3-kinase (PI3K)/protein kinase B (Akt), mammalian target or rapamycin (mTOR), mitogen-activated protein kinase (MAPK), signal transducer and nuclear factor kappa B (NF-κB) pathways and other pathways (Choi et al., 2020).

To date, three adiponectin receptors have been identified, including adiponectin receptor 1 (AdipoR1), adiponectin receptor 2 (AdipoR2), and T-cadherin (T-cad) (Yamauchi et al., 2003; Achari and Jain, 2017). AdipoR1 is predominantly expressed in skeletal muscle cells and acts as a high-affinity receptor for globular adiponectin and a low-affinity receptor for full-length adiponectin, whereas AdipoR2 is mainly expressed in hepatocytes and serves as a medium-affinity receptor for both full-length and globular adiponectin (Yamauchi et al., 2014). T-cad is mainly expressed in endothelial and smooth muscle cells, acting predominantly as a biologically active receptor that binds to adiponectin in diverse tissues and organs, such as in muscle, blood vessels, and the heart (Parker-Duffen et al., 2013; Clark et al., 2017). Adiponectin and its receptors are widely expressed in the hypothalamus, pituitary gland, and ovaries of humans, rats and pigs, as well as in the placenta and uterus of humans, mice and pigs, where they have been shown to functionally modulate the female reproductive system (Caminos et al., 2005; Kos et al., 2007; Rodriguez-Pacheco et al., 2007; Kim et al., 2011; Angelidis et al., 2013; Kiezun et al., 2013; Maleszka et al., 2014; Smolinska et al., 2014). As the characteristics of metabolic syndrome are associated with a number of reproductive disorders such as polycystic ovary syndrome (PCOS), gestational diabetes mellitus, preeclampsia, EMs, fetal growth restriction, and ovarian and endometrial cancers, their pathogenesis may be influenced by adiponectin (Barbe et al., 2019). AdipoR1 and AdipoR2 have been shown to be highly expressed in the normal human endometrium during the mid-secretory phase (i.e., during implantation), and adiponectin can induce the phosphorylation of AMPK, inhibit the expression of interleukin 1 beta (IL-1β), and promote the secretion of interleukin 6 (IL-6), interleukin 8 (IL-8), and monocyte chemoattractant protein 1 (MCP-1) in cultivated endometrial cells, suggesting that it may play both physiological and pathological roles within the endometrium (Takemura et al., 2006). In female mice, adiponectin may induce preimplantation embryo development and endometrial decidualization in an autocrine-, paracrine-, or endocrine-dependent manner (Kim et al., 2011). Furthermore, hypoadiponectinemia has been shown to be associated with an increased risk of hormone-dependent cancers, such as endometrial, ovarian, cervical, and breast cancers, as well as a poor prognosis (Zeng et al., 2015; Tsankof and Tziomalos, 2022).

## 4 Evidence for the association of adiponectin with EMs

Clinical studies, *in vitro* cell experiments, and animal experimental studies have provided evidence for the association between adiponectin and EMs. Clinical analysis has shown differences in adiponectin levels in patients with EMs. Moreover, studies have shown a decreased expression of adiponectin in patients with EMs; however, the findings are still controversial (Table 1). For example, Takemura et al. discovered a reduction in adiponectin levels in both the serum and peritoneal fluid (PF) of patients with EMs, with a particularly pronounced decrease in serum levels in those in the advanced stages of the disease (stage III/IV) (Takemura et al., 2005a,b). Similarly, another study reported lower serum levels of adiponectin in patients with EMs compared with that in healthy controls (Meng and Hao, 2020). However other researchers have observed no significant differences in the serum and PF levels of adiponectin between patients with infertility with and without concomitant pelvic EMs (Pandey et al., 2010). Others have reported no significant differential expression of adiponectin and AdipoRs in ectopic endometrial tissues of patients with ovarian EMs and patients with normal endometrial tissues who had undergone hysterectomy to treat cervical fibroids or carcinoma *in situ* (Choi et al., 2013). A meta-analysis published in 2021 that evaluated the results of 25 studies confirmed that although there was no significant association between the levels of adiponectin and disease severity, the patients with EMs did exhibit significantly lower levels of adiponectin and significantly higher levels of leptin compared to the concentrations in the control groups (Zhao Z. et al., 2021). Furthermore, a recent study that assessed adiponectin concentrations in the plasma, PF, and endometrioma fluids of 56 women with ovarian EMs reported that adiponectin levels were significantly lower in endometrioma fluids and PF than in serum, with a positive correlation between the concentrations in endometrioma fluids and PF (Wójtowicz et al., 2023). In addition, another group discovered that two polymorphisms in the adiponectin gene, rs2241766 and rs1501299, were associated with EMs susceptibility among women of childbearing age in Henan Province, China, and that the G mutation in the rs2241766 locus may affect splicing and modification of gene expression, resulting in the promotion of adiponectin secretion, which, in turn, induces AMPK phosphorylation and regulates its downstream pathway, thereby protecting against the development of EMs (Zhang et al., 2021). Both AdipoR1 and AdipoR2 are expressed in the endometrium, and their mRNA levels become significantly elevated in the mid-secretory phase (Takemura et al., 2006).

**TABLE 1:** Clinical studies on adiponectin levels in endometriosis.

References	Experiment	Control	Sample size (experiment vs control)	Location	Change (experiment vs control)
Takemura et al. (2005b)	Patients with endometriosis	Women of reproductive age without endometriosis	54:26	PF	↓
Takemura et al. (2005a)	Patients with endometriosis	Women of reproductive age without endometriosis	48:30	Serum	↓
Pandey et al. (2010)	Infertile patients with pelvic endometriosis	Infertile patients without pelvic endometriosis	15:35	PF	NS
				Serum	NS
Yi et al. (2010)	Patients with endometriosis	Patients without endometriosis who underwent surgical procedures for pelvic pain, infertility and ⁄ or other benign diseases	48:36	PF	NS
Huang et al. (2011)	Patients with endometriosis	Patients without endometriosis	57:30	PF	↓
	Patients with endometriosis (Ⅲ∼Ⅴ)	Patients with endometriosis (Ⅰ∼Ⅱ)	32:25		NS
Choi et al. (2013)	Patients with ovarian endometriosis	Age-matched patients who underwent hysterectomy because of myoma or carcinoma *in situ* of uterine cervix	44:42	Ectopic lesion	NS
Shah et al. (2013a)	Patients with endometriosis	Women of reproductive age without endometriosis	350:694	Blood	NS
Qin et al. (2014)	Patients with ovarian endometriosis	Healthy women	38:42	Serum	↓
Meng and Hao (2020)	Patients with endometriosis	Healthy women	30:30	Serum	↓
Wójtowicz et al. (2023)	Patients with ovarian endometriosis	Self-control	56	Plasma	9.8 μg/mL
					(7.3–13.1)
				PF	1.2 μg/mL
					(0.1–2.4)
				Endometrioma fluids	0.1 μg/mL
					(0.0–1.3)

In addition, the relationship between adiponectin and EMs has been explored using animal models and *in vitro* experiments. In studies on animal models, the number of stem cells in adipose tissue in mouse models of EMs decreased, and EMs changed the expression of multiple adipocyte metabolic genes, including adiponectin and leptin (Zolbin et al., 2019). *In vitro* cell experiments, Adiponectin has been shown to inhibit the proliferation of ectopic endometrial cells in a dose- and time-dependent manner while simultaneously increasing the expression of both AdipoR1 and AdipoR2 to inhibit EMs development; these findings suggest that adiponectin may serve as a beneficial factor that limits the pathogenesis of EMs (Bohlouli et al., 2016). Despite the plethora of studies conducted to date, the detailed regulatory role of adiponectin in the pathogenesis of EMs has yet to be elucidated, and further research is needed to deepen the understanding of the complex biological mechanisms through which adiponectin protects against EMs pathogenesis.

## 5 Possible associations of adiponectin with the pathogenesis of EMs

The formation of ectopic endometriotic lesions involves a series of complex events, including the adhesion, invasion, and angiogenesis of Endometrial cells to “take root, grow, and become sick.” First, due to an imbalance in immune function, ectopic endometrial cells are able to evade immune clearance (Symons et al., 2018). In addition, there is an imbalance between the proliferation and apoptosis of ectopic endometrial cells, which contributes to their survival in ectopic sites (Reis et al., 2013; Azam et al., 2022). After the adhesion of ectopic endometrial cells to ectopic sites, an inflammatory response is initiated, leading to increased secretion of pro-inflammatory mediators, and more inflammatory mediators are recruited to invade surrounding tissues (Wei et al., 2020; Artemova et al., 2021; Kapoor et al., 2021). Periodic bleeding of ectopic lesions and chronic inflammation activate the deposition and adhesion of fibrin and scar formation, leading to fibrogenesis of the lesion (Eming et al., 2014; Garcia et al., 2023). Simultaneously, the production of angiogenesis-related factors increases, promoting neoangiogenesis, providing nutrients for the survival and growth of ectopic lesions (Wei et al., 2020). In addition, ectopic endometrial cells also have changes in mitochondrial morphology and function, which helps to increase the energy supply to ectopic lesions and meet the growth needs of lesions (Ye et al., 2023). EMs is an estrogen-dependent disease in which estrogen and estrogen receptor (ER) levels are elevated in ectopic lesions (Burney and Giudice, 2012; Chantalat et al., 2020). Dysregulation of estrogen signal transduction causes ectopic endometrial cells to appear abnormal in cell proliferation and apoptosis, migration, invasion, angiogenesis and immune function, accompanied by increased inflammation and enhanced mitochondrial biosynthesis, which further promote lesion progression (Monsivais et al., 2016; Wu L. et al., 2019; Xu Z. et al., 2019; Marquardt et al., 2019; Qi et al., 2020; Kobayashi et al., 2021a). Therefore, EMs is a complex disease that is simultaneously affected by multiple factors. In addition to estrogen dependence, its pathogenesis mainly involves apoptosis, autophagy, inflammation, angiogenesis, fibrogenesis, and energy metabolism. Current research on the pathogenesis of EMs and the function of adiponectin have shown that adiponectin may play a special regulatory role in multiple biological processes related to the pathogenesis of EMs, such as apoptosis, autophagy, inflammatory response, angiogenesis, fibrogenesis, energy metabolism, and estrogen regulation. This aspect is noteworthy; hence, we have summarized the following important biological processes to reveal the mysterious relationship between EMs and adiponectin.

### 5.1 Apoptosis and autophagy

EMs is characterized by the invasive growth of active endometrial tissue at sites other than the uterus, and these events may be related to a disequilibrium between proliferation and apoptosis in ectopic endometrial cells. During these processes, these cells are less sensitive to apoptotic signals (Reis et al., 2013; Azam et al., 2022), therefore, ectopic endometrial cells can easily escape clearance and invade the peritoneum. Moreover, their proliferative activity is markedly increased (Jiang and Wu, 2012), leading to neovascularization and the establishment of endometrial implants, which leads to the development of the disease and further malignant progression. Compared to that of healthy controls, the expression level of the anti-apoptotic gene B-cell lymphoma 2 (Bcl-2) is significantly elevated in the ectopic endometrium of patients with EMs (Agic et al., 2009; Jiang R. et al., 2020), whereas that of the pro-apoptotic genes p53 and caspase-3 is decreased (Sang et al., 2019; Duan R. et al., 2020; Zhang and Zhao, 2023). Cyclin D1 and cyclin E2 are key regulatory proteins that control the transition from the G1 phase into the S phase during the cell cycle (Zabihi et al., 2023), and the expression of cyclin D1 mRNA has been shown to be significantly elevated in ectopic endothelial tissues compared to that in normal and eutopic endothelium (Pellegrini et al., 2012). Knockdown of cyclin D1 in human ectopic endometrial cells has been shown to significantly reduce their rate of proliferation while significantly increasing the number of cells in the G1/G0 phase (Hirakawa et al., 2017). Akt is a major protein kinase that exists downstream of PI3K and regulates the expression of various proteins associated with both cellular proliferation and apoptosis (Cinar et al., 2009; Revathidevi and Munirajan, 2019), and a recent study demonstrated that PI3K expression increases and Akt phosphorylation levels become elevated in the eutopic and ectopic endometrium of patients with EMs compared to the levels in healthy controls, indicating that the PI3K/Akt signaling pathway may play an active role in facilitating the establishment of ectopic endometrial tissue (Madanes et al., 2020).

Another important factor downstream of the PI3K/Akt pathway is mTOR, an evolutionarily conserved serine/threonine protein kinase (Driva et al., 2023). Researchers have reported an upregulation in mTOR signaling in endometriotic foci (Choi et al., 2014), and the protein is involved in the modulation of key pathophysiological processes such as proliferation, differentiation, apoptosis, autophagy, and decidualization of endometrial cells (Guo and Yu, 2019; Driva et al., 2022). Administration of the mTOR inhibitor everolimus was shown to promote apoptosis and inhibit the formation of endometriotic foci, making it a potential therapeutic option for the treatment of EMs (Kacan et al., 2017). In addition, it has been demonstrated that the MAPK signaling pathway is involved in the survival and proliferation of ectopic endometrial cells as well as various other processes including but not limited to invasion and metastasis, inflammation, and angiogenesis (Bora and Yaba, 2021; Zhang et al., 2023). In the late proliferative and early secretory stages, the phosphorylation level of p38-MAPK becomes significantly elevated in both eutopic and ectopic endometriotic tissues in patients with EMs compared to that in the normal endometrium (Cakmak et al., 2018), and aberrant activation of the MAPK signaling pathway can aggravate the severity of EMs (Jiang Y. et al., 2020; Bora and Yaba, 2021). Extracellular signal-regulated kinase (ERK), a member of the classical MAPK family (Samson et al., 2022), transmits extracellular signals following its activation, thereby promoting MAPK phosphorylation and regulating the expression of downstream effectors; thus, it plays a significant role in cell proliferation, differentiation, and apoptosis (Zhu et al., 2019; Bora and Yaba, 2021).

In humans, adiponectin has been reported to inhibit the proliferation of both normal and ectopic endometrial cells in a dose- and time-dependent manner (Bohlouli et al., 2013,^2016^), thereby playing a potentially important role in the pathological processes that lead to EMs. Additionally, other studies have shown that adiponectin can inhibit the proliferation of focal cells and exert pro-apoptotic effects in various diseases, including uterine fibroids as well as endometrial, ovarian, and breast cancers. The presence of both AdipoR1 and AdipoR2 has been confirmed in uterine leiomyoma cells, and adiponectin exerts a significant inhibitory effect on the proliferation of uterine Eker leiomyoma tumor 3(ELT-3) cells in rats (Wakabayashi et al., 2011; Strzałkowska et al., 2021). Adiponectin has been shown to decrease the expression of both cyclin D1 in the human endometrial adenocarcinoma cell lines HEC-1-A and KLE and cyclin E2 in the RL95-2 cell line by activating the AMPK signaling pathway, which led to the blockage of the cell cycle to inhibit the proliferation of cancer cells (Cong et al., 2007; Moon et al., 2011). In addition, adiponectin can mediate anti-proliferative and pro-apoptotic responses in endometrial cancer by inhibiting the activation of the Akt and ERK signaling pathways (Zhang et al., 2015). Administration of the AdipoR agonist AdipoRon has been shown to exert anti-tumor effects by inducing G1-phase cell cycle arrest and upregulating the expression of the pro-apoptotic gene caspase-3 via the activation of the AMPK and inhibition of mTOR signaling pathways, in human ovarian cancer cells (Ramzan et al., 2019). In the human breast cancer cell line MDA-MB-231, adiponectin was shown to inhibit cell growth by significantly reducing the expression of both cyclin D1 protein and the anti-apoptotic gene Bcl2, while simultaneously upregulating the expression of the pro-apoptotic genes p53 and Bax (Dos Santos et al., 2008; Delort et al., 2012). Furthermore, in MCF7 human breast cancer cells, adiponectin has been shown to induce anti-proliferative and pro-apoptotic responses by activating the AMPK signaling pathway and inhibiting the MAPK signaling pathways (Dieudonne et al., 2006).

While the pro-apoptotic effects of adiponectin can protect against the development of various diseases, some studies have also demonstrated its anti-apoptotic activity. For example, in a mouse model of sepsis, adiponectin has been shown to inhibit lipopolysaccharide (LPS)-induced cardiomyocyte apoptosis by downregulating connexin 43 (Cx43) expression and activating the PI3K/AKT signaling pathway to protect myocardial function (Liu et al., 2021). AdipoRon administration reduces high glucose-induced oxidative stress and apoptosis and ameliorates endothelial dysfunction via the activation of the AMPK/PPAR-α pathway, thereby exerting a nephroprotective effect in diabetic nephropathy (Kim Y. et al., 2018). In rheumatoid arthritis (RA), adiponectin has been shown to promote the proliferation and differentiation of B cells by inducing the activation of the PI3K/Akt and activator of transcription 3 (STAT3) signaling pathways, exacerbating RA development (Che et al., 2021). Additionally, in early pregnancy in pigs, adiponectin was shown to induce porcine uterine luminal epithelial cell proliferation while inhibiting apoptosis through the activation of the PI3K/Akt and MAPK signaling pathways, enhancing uterine tolerance to embryonic implantation (Lim et al., 2017). It is important to note, however, that some studies have also demonstrated no effect of adiponectin on apoptosis (Arditi et al., 2007; Pfeiler et al., 2008); thus, its role in such processes remains controversial, and its effects may be related to the disease, the source of adiponectin, as well as differences in conformations, concentrations, and treatment durations between studies (Sun and Chen, 2010).

Apoptosis is not the sole mechanism of endometrial cell death (Choi et al., 2015). Autophagy, another form of programmed cell death, involves a series of catabolic processes that depend on the lysosomal degradation of proteins and cytoplasmic organelles, which are closely related to cellular proliferation and apoptosis (Popli et al., 2022). The expression levels of autophagy-related genes such as microtubule-associated protein light chain 3 (LC3) and Beclin-1 have been shown to be reduced in the serum, PF, and eutopic endometrial tissue of patients with EMs compared with the levels in healthy controls (Sui et al., 2018), and the level of expression of autophagy-related genes (LC3B-II) was significantly reduced in ectopic endometrium compared with that in the eutopic endometrial tissues of patients with EMs (Li et al., 2018). Inhibition of autophagy can promote the expression of inflammatory cytokines, trigger inflammation and immune responses leading to autoimmune damage, enable endometrial cells to evade immune surveillance, and ultimately promote ectopic growth and implantation of ectopic endometrial cells in the peritoneal cavity (Ji et al., 2022). Autophagy and apoptosis are not two independent processes but two crosstalk mechanisms. Autophagy promotes the phagocytosis of apoptotic bodies and degradation of lysosomes (Mariño et al., 2014); therefore, decreased autophagic activity in ectopic and eutopic endometrial cells would lead to a decrease in apoptosis (Choi et al., 2014; Yu et al., 2016). As mTOR is a major negative regulator of autophagy, several studies have reported that cellular autophagy can be induced by inhibiting the mTOR signaling pathway in ectopic endometrial cells (Choi et al., 2015; Xu H. et al., 2019; Huang et al., 2023). In addition, in mouse models of EMs, in addition to inhibiting autophagy, the upregulation of mTOR in EMs tissues promoted the survival of ectopic endometrial cells (Yang et al., 2017). In terms of other signaling pathways, AMPK activation results in the downregulation of PI3K/Akt/mTOR signaling, leading to decreased phosphorylation of the protein autophagy-related 13 (ATG13) (Yang et al., 2017; Assaf et al., 2022; Ge et al., 2022) and an increase in the level of the unc-51-like autophagy activating kinase 1 (ULK1) complex formed by the interaction of ATG13 with ULK1, FAK family kinase-interacting protein of 200 kDa (FIP200), and autophagy-related 101 (ATG101); this complex is an important factor in the initiation of autophagy (Yang et al., 2017). Administration of AdipoRon can activate AMPK/mTOR signaling to promote autophagy (Duan Z. X. et al., 2020) and reduce the dysregulated expression of proteins involved in the autophagy-lysosomal pathway (He et al., 2021). Moreover, researchers have shown that adiponectin can induce autophagy in breast cancer cells by activating the AMPK-ULK1 axis mediated by serine/threonine kinase 11 (STK11)/liver kinase B1 (LKB1), which, in turn, promotes breast cancer cell apoptosis (Chung et al., 2017).

It must be acknowledged that the regulatory effect of adiponectin on autophagy is not unilateral, as others have shown that adiponectin inhibits autophagy and reduces angiogenesis in the choroidal retinal endothelial cell line RF/6A in monkeys through activation of the PI3K/AKT/mTOR pathway (Li et al., 2019). Adiponectin can protect against hypoxia/reperfusion injury-induced cardiomyocyte damage by inhibiting autophagy as well, an effect that may be related to the inhibition of the AMPK/mTOR/UKL1/Beclin-1 pathway (Guo et al., 2022).

Although the role of adiponectin in the regulation of apoptosis and autophagy may be bidirectional, many studies have provided ample evidence that it plays a role in protecting against the development of various diseases and may serve as a potentially therapeutic strategy. Although relevant studies have confirmed the role of adiponectin in the inhibition of endometrial cells proliferation in EMs, whether the mechanism of action is related to the regulation of apoptotic genes and signal pathways such as AMPK, MAPK, PI3K/Akt/mTOR and so on, more research is needed to explain.

### 5.2 Angiogenesis

The shed endometrial cells undergo migration, adhesion, invasion and implantation with retrograde menstruation to form ectopic lesions, during which massive neovascularization occurs and a new blood supply is established to provide nourishment for the survival and growth of the lesions (Wei et al., 2020). Therefore, angiogenesis is considered a critical step in the establishment and persistence of endometriotic lesions. The results of studies involving animal models have suggested that anti-angiogenic therapies substantially reduce the size of ectopic lesions (Liu et al., 2016); in EMs, angiogenesis is regulated by a multitude of factors and pathways involved in those processes. For example, vascular growth factors may play an essential role in promoting the growth and differentiation of ectopic endometrial tissue, with vascular endothelial growth factor (VEGF) being the most critical. Members of the VEGF family, which includes VEGF-A, VEGF-B, VEGF-C, VEGF-D, and VEGF-E, as well as placental growth factors, have been shown to increase vascular permeability, accelerate vascular endothelial cell migration and proliferation, and promote angiogenesis (Ferrara and Adamis, 2016), and VEGF and its signaling pathway are considered the optimal targets for anti-angiogenic and anti-tumor therapies for a variety of cancers (Potente et al., 2011; Gao et al., 2020). VEGF expression was elevated in healthy controls, in the eutopic endometrium of patients with Ems, and in ectopic lesions sequentially (Di Carlo et al., 2009), and elevated VEGF expression in the serum and PF of patients with EMs may serve as a secondary diagnostic indicator of the disease (Bourlev et al., 2010; Li et al., 2020). VEGF expression in EMs is regulated by multiple factors and associated pathways, and it plays a pivotal regulatory role in neovascularization.

Adiponectin is an important regulator of angiogenesis, exerting its effect through the regulation of VEGF. In prostate cancer cells, adiponectin can prevent neovascularization by suppressing VEGF-A secretion (Gao et al., 2015), and adiponectin treatment has been shown to downregulate VEGF-B and VEGF-D mRNA expression while increasing the serum concentration of the anti-angiogenic factor IL-12 in mouse colon cancer cells (Moon et al., 2013). Other important factors to consider are the matrix metalloproteinases (MMPs), which are involved in the degradation of the extracellular matrix and the induction of vascular endothelial cell proliferation and angiogenesis (D’Amico et al., 2020). MMP-2 expression has been shown to be significantly elevated in the ectopic foci, PF, and serum of patients with EMs (Sui et al., 2018). And adiponectin inhibits angiogenesis by downregulating MMP expression. For example, in liver cancer tissues in mice, adiponectin has been shown to downregulate the mRNA expression of VEGF and MMP-9, thereby inhibiting tumor angiogenesis (Man et al., 2010). Furthermore, in human renal carcinoma cells, adiponectin can activate AMPK via binding to AdipoR1, inhibiting the mTOR signaling pathway and suppressing the production of VEGF, MMP-2, and MMP-9 (Kleinmann et al., 2014). There have also been reports that adiponectin can inhibit the proliferation and migration and angiogenesis of endothelial cells (Dubois et al., 2013; Li et al., 2019; Palanisamy et al., 2019). Such an inhibitory effect may indirectly hinder angiogenesis of ectopic lesions. Additionally, adiponectin can induce endothelial apoptosis, which occurs during angiogenesis (Watson et al., 2016), by activating caspase-8, thereby leading to the activation of caspase-3 or caspase-9 (Bråkenhielm et al., 2004).

The inhibitory role of adiponectin in angiogenesis has been demonstrated in multiple types of cancer, including fibrosarcoma (Bråkenhielm et al., 2004), hepatocellular carcinoma (Man et al., 2010), renal cell carcinoma (Kleinmann et al., 2014), basal cell breast cancer (Dubois et al., 2013), and prostate cancer (Gao et al., 2015). However, several other studies have shown that adiponectin can *enhance* angiogenesis by upregulating the expression of pro-angiogenic factors in human umbilical vein endothelial cells (HUVECs) and human microvascular endothelial cells (HMECs) (Adya et al., 2012; Nigro et al., 2021), and it has been shown to promote angiogenesis in chondrosarcoma (Lee et al., 2015), invasive colon cancer (Cai et al., 2016), and ovarian cancer (Ouh et al., 2019), possibly via the regulation of CXC motif chemokine ligand 1 (CXCL1), VEGF, and AMPK. Adiponectin enhances CXCL1 secretion, which, in turn, promotes VEGF secretion and angiogenesis (Kiefer and Siekmann, 2011) in various cancers, including colon (Cai et al., 2016) and ovarian cancer (Ouh et al., 2019). AMPK is a key molecule involved in the regulation of biological energy metabolism, and inhibition of its activity significantly attenuates VEGF secretion (Fisslthaler and Fleming, 2009). In HUVECs and HMEC-1 cells, adiponectin has been shown to induce angiogenesis by activating the AMPK/Akt pathway and phosphorylating endothelial nitric oxide synthase (eNOS) (Ouchi et al., 2004; Adya et al., 2012), which is various with the findings of another study that showed angiogenesis was inhibited by adiponectin (Dubois et al., 2013). In addition, adiponectin may play a pro-angiogenic protective role in ischemic tissue injury. For example, the overexpression of adiponectin was shown to induce the upregulation of VEGF mRNA expression within ischemic regions of the brains of mice, enhancing focal angiogenesis (Shen et al., 2013). Another study showed that recovery following ischemic reperfusion injury of a limb in mice was accelerated by giving adenovirus-mediated adiponectin and exogenous adiponectin administration stimulated angiogenesis in response to ischemic stress by activating the AMPK pathway (Shibata et al., 2004).

Collectively, angiogenesis in EMs has similar features to pathological angiogenesis in tumor growth and metastasis, being co-regulated by multiple factors. Existing studies have shown that adiponectin plays a role in the inhibition of angiogenesis in many oncological diseases; however, it is not clear whether adiponectin has a beneficial role in EMs by regulating signaling pathways such as AMPK and cytokines such as VEGF and MMPs to promote endothelial cell apoptosis, inhibit endothelial cell migration and thus inhibit angiogenesis. However, for this conjecture, we are currently inconclusive, because the regulation of adiponectin-mediated angiogenesis is bidirectional, especially in oncological diseases, with different modes of action. This paradoxical phenomenon could be due to variability between studies in terms of the differences in cell types, *in vivo* and *in vitro* experimental methodologies, and the physiological and pathological conditions in which angiogenesis is observed.

### 5.3 Inflammatory processes

Chronic inflammation and immune dysregulation are essential microenvironment and pathophysiological features of EMs (Zhang et al., 2018; Encalada Soto et al., 2022). Due to the dysregulation of immune mechanisms, ectopic endometrial cells evade immune clearance to survive, adhere, and invade ectopic sites (Symons et al., 2018). Subsequently, during the formation of endometriotic lesions, the secretion of a large number of pro-inflammatory cytokines is increased. Inflammatory cells are recruited to the lesion site, which secrete a variety of inflammatory mediators to form an inflammatory microenvironment. These mediators counteract inflammatory cells and factors, leading to the recruitment of more inflammatory cells to the lesion site, forming a vicious circle in which these immune molecules not only promote cell proliferation and adhesion but also contribute to the cell evasion of immunosurveillance, further exacerbating the invasion of ectopic endometrial cells and promoting lesion formation and progression (Wei et al., 2020; Artemova et al., 2021; Kapoor et al., 2021). Numerous studies have shown that the lymphocytes present within the peritoneal cavity in EMs are predominantly macrophages (Bacci et al., 2009; Shao et al., 2016; Ramírez-Pavez et al., 2021), which are broadly classified into two main phenotypes, including the pro-inflammatory M1-type and the anti-inflammatory M2-type (Laskin et al., 2011). M1 macrophages predominantly secrete pro-inflammatory factors such as tumor necrosis factor alpha (TNF-α), IL-6, and IL-1β (Gordon, 2003,^2007^), all of which have been shown to be expressed at elevated levels in the PF, serum, and ectopic lesions of patients with EMs (Bergqvist et al., 2001; Richter et al., 2005; Cho et al., 2007; Wang F. et al., 2018; Wang X. M. et al., 2018; Jaeger-Lansky et al., 2018; Volpato et al., 2018). M2 macrophages mainly secrete transforming growth factor beta (TGF-β), interleukin-10 (IL-10), and other anti-inflammatory cytokines, while inhibiting the production of pro-inflammatory cytokines (Wang et al., 2019; Yao et al., 2019; Gao et al., 2022). In endometriotic lesions, M2-type macrophages are predominant (Bacci et al., 2009; Hogg et al., 2020); however, their numbers become significantly reduced throughout the menstrual cycle in patients with EMs compared with the changes observed in healthy controls in the same time period (Takebayashi et al., 2015). There have been reports suggesting that anti-inflammatory factors may play a dual role in the later stage of EMs, as they can promote the immune escape of ectopic endometrial cells and induce inflammation, while also inhibiting inflammatory responses and reducing disease activity; however, further studies are required to establish a better understanding of their roles in EMs (Zhou et al., 2019). What is known is that NF-κB, a major regulator of inflammation and immune responses, can stimulate LPS-induced pro-inflammatory cytokine expression in macrophages (Monaco and Paleolog, 2004). NF-κB is overactive in endometriotic lesions, enhancing the proliferation, adhesion, migration, and invasive ability of ectopic endometrial cells (Liu et al., 2022).

Reduced serum levels of adiponectin have been reported to be associated with chronic inflammation in various metabolic diseases, including type 2 diabetes, obesity, atherosclerosis, and non-alcoholic fatty liver disease, where the anti-inflammatory effects of adiponectin have been demonstrated (Fantuzzi, 2008; Achari and Jain, 2017; Choi et al., 2020). The anti-inflammatory effects of adiponectin are mainly mediated via the targeting of macrophages (Tsatsanis et al., 2005; Ohashi et al., 2014; Fang and Judd, 2018), which can suppress the growth of myelomonocytic progenitor cell as well as the function of mature macrophages, hindering their phagocytotic ability and lowering TNF-α production, as well as reducing the expression levels of class A scavenger receptors, as well as reducing the expression levels of class A scavenger receptors (Yokota et al., 2000; Ouchi et al., 2001). Adiponectin inhibits the progression of various metabolic and cardiovascular diseases by promoting the transition of macrophages from the pro-inflammatory M1 phenotype to the anti-inflammatory M2 phenotype (Ohashi et al., 2010). In humans, the adiponectin-induced differentiation of monocytes into anti-inflammatory M2-type macrophages is mediated via PPAR-α, and these M2 macrophages inhibit the secretion of pro-inflammatory molecules from M1 macrophages, which may contribute to the stability of atherosclerotic plaques (Lovren et al., 2010). Although adiponectin can affect macrophage function through multiple other signaling pathways as well, the main targets are the AdipoR1/Toll/NF-κB and AdipoR2/IL-4/STAT6 signaling pathways, which inhibit effects on macrophage activation to the M1 phenotype and promote macrophage polarization toward the M2 phenotype, respectively (Yamaguchi et al., 2005; Mandal et al., 2011; Wang N. et al., 2014). Other researchers have discovered that adiponectin can attenuate LPS-induced TNF-α and IL-6 production by macrophages while upregulating the expression of anti-inflammatory IL-10 (Wulster-Radcliffe et al., 2004). Several studies have demonstrated that adiponectin expression is also negatively regulated by pro-inflammatory TNF-α and IL-6, which may contribute to the presence of hypoadiponectinemia in inflammatory diseases (Tilg and Wolf, 2005; Brezovec et al., 2021). Furthermore, adiponectin effectively inhibits the activation of the NF-κB pathway by suppressing the expression of the NF-κB nuclear protein p65, which, in turn, reduces the expression of NF-κB-regulated pro-inflammatory factors and potently attenuates the inflammatory response to atherosclerosis (Wang et al., 2016). Within the endometrium, adiponectin plays a similar anti-inflammatory role by stimulating the phosphorylation of AMPK in Endometrial cells and inhibiting the IL-1β-induced secretion of the pro-inflammatory factors IL-6, IL-8, and MCP-1 (Takemura et al., 2006). These anti-inflammatory effects of adiponectin in Endometrial cells may be associated with endometrium-related events, such as endometrial implantation and the pathogenesis of EMs.

Although adiponectin is generally considered an anti-inflammatory adipokine, at high expression levels, it is positively correlated with the clinical progression of systemic autoimmune rheumatic diseases (SARDs), which are typically accompanied by high levels of inflammation; paradoxically, some studies have reported low expression levels of adiponectin in SARDs, with low levels of inflammation and a negative correlation with disease progression (Brezovec et al., 2021). Some recent studies have also shown that adiponectin can act as an inducer of pro-inflammatory factors and that elevated levels of adiponectin may exacerbate the inflammatory response associated with various autoimmune diseases such as RA, osteoarthritis (OA), systemic lupus erythematosus, and inflammatory bowel diseases (Choi et al., 2020; Brezovec et al., 2021). Others have also found that HMW adiponectin promotes the secretion of TNF-α and IL-6 by fibroblasts from fibroblastic synoviocytes in patients with RA, aggravating the severity of inflammation (Kontny et al., 2015; Li et al., 2015; Liu et al., 2020). In OA, adiponectin has been shown to promote IL-8 secretion in osteoblasts, thereby affecting osteophyte development (Junker et al., 2017). In addition, others have reported than adiponectin can enhance the mRNA expression and protein secretion of IL-8 in human colonic epithelial and macrophage cell lines (Peng et al., 2018). Furthermore, globular adiponectin was shown to be capable of activating the NF-κB pathway and upregulating the expression of pro-inflammatory cytokines in human placenta and adipose tissue (Lappas et al., 2005), HUVECs (Hattori et al., 2006), and RAW264.7 murine macrophages (Lee et al., 2018).

Adiponectin can participate in the inflammatory response of many diseases by regulating macrophage polarization and proliferation and signaling pathways, such as NF-κB and AMPK, as well as the expression of related inflammatory mediators. However, depending on its isoforms and effector tissues, adiponectin may exert differential effects in various physiological processes (Choi et al., 2020), and its bidirectional regulatory function in inflammatory diseases may be related to the severity and stage of disease progression. Further studies is requires to determine whether adiponectin could inhibit the inflammatory response in EMs by regulating signaling pathways, such as NF-κB and AMPK, as well as the expression of related inflammatory mediators and macrophage polarization and proliferation. Moreover, whether adiponectin plays different roles, such as inhibiting or promoting inflammation depending on the severity and progression of EMs, needs to be established.

### 5.4 Fibrogenesis

Over time, ectopic endometrial cells gradually form ectopic lesions through migration, invasion, proliferation, and growth. The lesions undergo repeated bleeding and injury, triggering a recurrent cycle of inflammatory responses and tissue repair (Garcia et al., 2023). Dysregulated repair mechanisms result in excessive accumulation of extracellular matrix, leading to the formation of adhesions, permanent scarring, and the disruption of tissue structure, causing organ dysfunction and ultimately resulting in fibrosis of the endometriotic tissue. Immune cells migrate to the fibrotic region, releasing inflammatory factors, increasing local inflammatory response, and exacerbating the growth and invasion of endometrial cells (Izumi et al., 2018). Fibrosis is inherent in all forms of EMs, and it is associated with painful symptoms, altered tissue function, and impaired fertility in patients with EMs (Eming et al., 2014; Garcia et al., 2023). Fibrosis of ectopic lesions in EMs is an abnormally proliferative disorder that occurs with repeated rupture of the ectopic cyst wall and outflow of the cyst contents, which irritate the adjacent tissues, followed by a localized inflammatory response and fibrosis formation. The histopathological features of ectopic endometriotic lesions are dominated by a large amount of fibrotic tissue, in addition to the endothelial glands and mesenchymal stromal cells (Liu et al., 2018). Fibrogenesis is an important process in the development of EMs; therefore, probing the mechanism underlying fibrogenesis in EMs and finding specific and effective therapeutic targets to inhibit fibrogenesis may be a potential methodology for the treatment of EMs (Vigano et al., 2018). The process of fibrosis in EMs requires the involvement of a variety of cytokines and cells, including macrophages, myofibroblasts, platelets (Kendall and Feghali-Bostwick, 2014; Weiskirchen et al., 2019; Yang and Plotnikov, 2021). Of particular importance is TGF-β, a multipotent cytokine that regulates cell growth, development, differentiation, proliferation, immunomodulation, the maintenance of homeostasis, and fibrosis via its downstream signal transduction pathway (Morikawa et al., 2016). TGF-β1 is a major regulator of tissue repair as well as fibrosis, initiating the collagen accumulation program and mediating the fibrotic process through multiple signaling pathways (Kim K. K. et al., 2018; Hu et al., 2018; Lodyga and Hinz, 2020; Ye and Hu, 2021). Significantly elevated levels of TGF-β1 have been reported in the serum, PF, and ectopic endometrial tissue of patients with EMs (Chang et al., 2017; Gueuvoghlanian-Silva et al., 2018; Sikora et al., 2018). In ovarian EMs, these ectopic endometrial cells secrete TGF-β1 and activate the Smad signaling pathway to disrupt the extracellular matrix and promote fibrosis in the tissue surrounding the ectopic lesions (Shi et al., 2017). The degree of fibrosis in human ovarian EMs (Ding et al., 2020) and superficial peritoneal EMs (Ibrahim et al., 2019) was positively correlated with the expression levels of alpha-smooth muscle actin (α-SMA). Elevated α-SMA expression is a signifier of the transformation of fibroblasts into myofibroblasts, which is a key process of fibrosis (Malmström et al., 2004; Duan et al., 2018).

In recent years, many studies have demonstrated that adiponectin plays a role in preventing fibrosis in a wide range of organs and tissues and that it may represent a potential therapeutic target for the treatment of fibrosis. The anti-fibrotic effects are mediated through the altered signaling of various pathways such as AMPK, PPAR, and TGF-β1/Smad, inhibiting the differentiation of fibroblasts to myofibroblasts and reducing the production as well as the deposition of extracellular matrix. Numerous studies have investigated its mechanism of action in fibrotic diseases of the kidneys (Jing et al., 2020; Zhao D. et al., 2021), liver (Dong et al., 2015; Udomsinprasert et al., 2018), heart (Fujita et al., 2008; Qi et al., 2014), lungs (Kökény et al., 2021; Wang et al., 2022), and skin (Wang et al., 2023), among other organs and systems, confirming its protective effects. Adiponectin was found to be capable of ameliorating tubulointerstitial fibrosis and suppressing angiotensin II-induced secretion of TGF-β1 and fibronectin in human renal mesangial cells, thereby reducing extracellular matrix synthesis (Tan et al., 2015). Additionally, α-SMA expression was shown to be downregulated by adiponectin in the renal cortex of mice with progressive renal injury induced by the administration of deoxycorticosterone acetate and angiotensin II, as well as in the lung tissues of mice with bleomycin-induced idiopathic pulmonary fibrosis (Tian et al., 2018; Wang et al., 2022). Another showed that the administration of the AdipoR agonists JT002, JT003, and JT004 significantly reduced the protein expression level of α-SMA in hepatic stellate cells and slowed the progression of hepatic fibrosis in mice (Xu et al., 2020).

While most studies to date have reported a protective effect of adiponectin, a few have shown the opposite effect, with adiponectin driving the pathogenesis of fibrosis. For example, one group showed that in mouse bone marrow-derived macrophages, adiponectin activated the AMPK signaling pathway in a time- and dose-dependent manner, induced α-SMA expression, enhanced the production and deposition of extracellular matrix, and promoted the cells’ transformation into fibroblasts, which accelerated renal interstitial fibrosis (Yang et al., 2013). Several other researches have suggested an association between high serum levels of adiponectin and renal functional decline (Panduru et al., 2015; Zha et al., 2017).

Low levels of adiponectin are associated with fibrosis. Serum adiponectin levels in patients with non-alcoholic fatty liver disease are an independent predictor of advanced fibrosis and are negatively correlated with the stage of liver fibrosis (Savvidou et al., 2009). A study showed that serum adiponectin levels in patients with hypertension significantly correlated negatively with biomarkers of myocardial fibrosis (Balmaceda et al., 2020). However, some controversial views have been reported (Arvaniti et al., 2008; Korah et al., 2013; Yan et al., 2013). Adiponectin can inhibit fibrogenesis by regulating signaling pathways, such as TGF-β1/Smad, inhibiting α-SMA expression, and reducing the production and deposition of the extracellular matrix. Adiponectin acts as an important protective factor in fibrotic diseases of the liver, kidneys, heart, lungs, and skin, and low serum adiponectin levels may be an indicator of the progression of fibrogenesis. TGF-β1 is highly expressed in EMs, and activation of the Smad signaling pathway by TGF-β1 can promote fibrosis in tissues surrounding ectopic endometriotic lesions. EMs-associated fibrosis is a relatively new area of research, it is necessary to determine whether the low adiponectin levels in patients with EMs are related to the severity of fibrosis in EMs or whether adiponectin plays a protective role in fibrosis of EMs by modulating signaling pathways, such as TGF-β1/Smad.

### 5.5 Energy metabolism

The peritoneal microenvironment undergoes significant alterations in patients with EMs; these changes are characterized by imbalances in inflammation, hypoxic conditions, and increased oxidative stress (McKinnon et al., 2016; Ito et al., 2017; Lin et al., 2018; Wu M. H. et al., 2019), which can adversely affect mitochondrial respiration and dysfunctional activity in ectopic endometrial cells, leading to metabolic abnormalities (Atkins et al., 2019; Kobayashi et al., 2021b). In order to adapt to the complex environment and meet the energetic needs of proliferating lesions, ectopic endometrial cells regulate the morphology and function of mitochondria by transforming metabolic processes and activating various signaling pathways, such as that of AMPK, promoting mitochondrial energy production and increasing the supply of available energy to meet the growing requirements of diseased tissues; ultimately, these changes promote the migration, invasion and proliferation of ectopic endometrial cells and create an environment that is conducive to the progression of the lesions (Kasvandik et al., 2016; Kobayashi et al., 2021b; Assaf et al., 2022).

Mitochondria are the organelles that produce the energy required to sustain cellular activity by constantly undergoing repeated cycles of fusion and division (Ye et al., 2023). Mitochondrial dysfunction and reduced energy metabolism have been reported in granulosa and ectopic endometrial cells of patients with EMs (Hsu et al., 2015; Kobayashi et al., 2023), and the altered mitochondrial dynamics and morphology increase the survivability of ectopic endometrial cells in hypoxic and oxidative-stress-related environments, thereby facilitating EMS progression (Ye et al., 2023). Oxidative stress is defined as an imbalance between oxidants, such as reactive oxygen species (ROS), and antioxidants, such as the enzyme superoxide dismutase (SOD). ROS are chemically reactive substances that mediate redox signaling and regulate cellular functions; in patients with EMs, they are involved in maintaining the uterine proliferative phenotype of endometrial cells, increasing the likelihood of ectopic tissue invasion and implantation (Cacciottola et al., 2021). It has been shown that the production of endogenous ROS is increased above normal levels in ectopic endometrial cells of patients with EMs (Ngô et al., 2009); this is accompanied by elevated concentrations of oxidative stress markers in the PF, such as advanced oxidation protein products (Santulli et al., 2015). As a consequence of long-term exposure to oxidative stress, the mitochondria of ectopic endometrial cells promote ROS secretion through an elongation mechanism, further exacerbating oxidative stress and forming a vicious circle that promotes the progression of lesions in EMs (Assaf et al., 2022). Plasma SOD activity has been shown to be reduced in patients with EMs, which is an indicator that the antioxidant capacity has been compromised (Prieto et al., 2012).

Maintaining systemic energy homeostasis is an essential function of most adipocyte-derived hormones. Adiponectin is an adipocyte-derived hormone that improves insulin sensitivity in the liver and skeletal muscle. Studies have shown that in addition to insulin sensitization, adiponectin plays an important role in maintaining systemic energy homeostasis (Wang and Scherer, 2016; Fang and Judd, 2018); it can also promote mitochondrial biogenesis and oxidative metabolism in skeletal muscles of both animals and humans (Lee and Shao, 2014), protect the morphology and function of mitochondria, facilitate the restoration of mitochondrial antioxidant capacity, and protect against oxidative damage following traumatic brain injury (Zhang et al., 2022). Another study demonstrated that adiponectin can also prevent neuroinflammation associated with mitochondrial damage, thereby regulating senescence in the brain (He et al., 2023). Furthermore, adiponectin can inhibit oxidative stress by regulating the balance between ROS and SOD, counteracting obesity-related metabolic changes and cardiovascular diseases and protecting the vascular endothelium and myocardium from tissue damage (Matsuda and Shimomura, 2014). Mechanistically, adiponectin was shown to alleviate LPS-induced oxidative stress during the pre-differentiation of adipocytes through modulation of the peroxisome proliferator-activated receptor gamma (PPAR-γ)/Nnat/NF-κB axis, significantly reducing the concentration of ROS and increasing the level of SOD in cells (Yang et al., 2019). It was also shown to inhibit oxidative stress by enhancing SOD activity in the serum of homozygous apolipoprotein E-deficient (ApoE −/−) mice, thereby reducing the formation of atherosclerotic plaques (Wang X. et al., 2014). In mouse hepatocytes and podocytes, adiponectin was shown to inhibit palmitic acid-induced ROS secretion and protect against liver and kidney injury (Dong et al., 2020; Xu et al., 2021).

However, a small number of studies have reported contradictory findings; for example, one group showed that adiponectin inhibits SOD activity and promotes ROS secretion in rat RINm insulinoma cells, but without reaching pathological concentrations (Chetboun et al., 2012), and others have reported that full-length adiponectin inhibits ROS production in human phagocytes, whereas globular adiponectin exerts the opposite effect (Chedid et al., 2012). The discrepant results of these studies suggest that adiponectin can modulate oxidate stress in a differential manner depending on the isoform.

Studies have shown that adiponectin can protect the morphology and function of the mitochondria, regulate the balance of ROS and SOD, and inhibit oxidative stress. Increased oxidative stress, mitochondrial dysfunction, and other abnormalities in energy metabolism in EMs increase the survival of ectopic endometrial cells; therefore, adiponectin may regulate energy metabolism in Endometrial cells in the microenvironment of the ectopic lesions to affect the ability of Endometrial cells to proliferate, migrate, and invade ectopic sites, and thus play a role in the pathogenesis of EMs; however, the specific mechanism requires further research.

### 5.6 Synthesis, metabolism, and effects of estrogen

EMs is an estrogen-dependent disease (Soares et al., 2012) and is characterized by estrogen-dependent growth of the ectopic endometrium and increased local estrogen secretion (Mori et al., 2019). Estrogen is mainly secreted by the ovarian granulosa and endothelial cells and includes three main types: estrone, estradiol (E2), and estriol, of which E2 has the highest expression and plays the greatest role (Mahboobifard et al., 2022). Aromatase P450, an important enzyme in the synthesis of estrogen, catalyzes the conversion of androstenedione and testosterone produced in ovarian follicular cells to estrone and E2 in ovarian granulosa cells, and its expression is increased in endometriotic lesions (Peitsidis et al., 2023). Aromatase inhibitors, such as anastrozole, letrozole, and exemestane, may be effective agents for the treatment of EMs, as they have the potential to control the symptoms associated with EMs in cases where no therapeutic response was achieved with an initial pharmacological intervention (Soares et al., 2012; Peitsidis et al., 2023). Estrogen plays a biological role by binding to ER. ERα expression is downregulated, and ERβ expression is abnormally high in ectopic endometrial cells compared with normal Endometrial cells. Moreover, ERβ can directly inhibit ERα expression (Monsivais et al., 2014; Yilmaz and Bulun, 2019). Localized high estrogenic environment and abnormally high ERβ levels promote ectopic endometrial cells proliferation, adhesion, and angiogenesis, as well as upregulated expression and release of pro-inflammatory factors, leading to immune dysfunction and enhancement of mitochondrial biosynthesis, which provides favorable conditions for lesion progression (Monsivais et al., 2016; Wu L. et al., 2019; Xu Z. et al., 2019; Marquardt et al., 2019; Qi et al., 2020; Kobayashi et al., 2021a).

Serum HMW adiponectin is negatively correlated with E2 concentrations in healthy premenopausal women (Merki-Feld et al., 2011), and this negative correlation may be related to the strong association between adiponectin and sex hormone-binding globulin (SHBG) (Tworoger et al., 2007). There is a significant positive correlation between serum adiponectin levels and SHBG levels (Tworoger et al., 2007; Wildman et al., 2013). SHBG can bind to estrogen in the plasma and directly regulate the bioavailability of estrogen and its access to target cells (Fortunati et al., 2010), whereas increased insulin resistance leads to inhibition of SHBG expression, which in turn leads to an increase in the concentration of biologically active estrogen (Hammond et al., 2008; Caselli, 2014; Winters et al., 2014). Adiponectin may counteract the inhibitory effect of SHBG by ameliorating insulin resistance. In addition, AdipoRon can activate the AMPK and PPAR-α pathways in human luteinized granulosa cells, down-regulating cyclic adenosine monophosphate production and aromatase protein expression, thereby reducing E2 production (Grandhaye et al., 2021). Similarly, Tao et al. found that in polycystic ovary syndrome disease, adiponectin downregulates P450 aromatase expression in human luteal granulosa cells and human chorionic gonadotropin-induced E2 synthesis, at least in part, through the activation of PPAR-α (Tao et al., 2019). A large number of studies have now demonstrated the correlation between low adiponectin levels and an increased risk of developing estrogen-dependent diseases (including cervical, endometrial, and breast cancers, as well as uterine fibroids) (Gelsomino et al., 2019; Strzałkowska et al., 2021). For example, Vivian et al. found that adiponectin inhibits the effects of estrogen on breast cancer cell proliferation by decreasing aromatase activity and ER mRNA levels (Morad et al., 2014). Low adiponectin levels are present in female patients with uterine fibroids, and adiponectin may inhibit smooth muscle tumor growth by lowering estrogen levels through the inhibition of the E2/ERα and insulin-like growth factor 1/insulin-like growth factor 1 receptor pathways (Strzałkowska et al., 2021).

The above studies revealed that adiponectin influences the synthesis, metabolism, and effects of estrogen by inhibiting aromatase expression and estrogen production through the regulation of AMPK and PPAR-α signaling pathways. Adiponectin may play a role in estrogen-dependent diseases by influencing insulin- and estrogen-dependent pathways. Studies have shown that there are high estrogen and low level of adiponectin expression in ectopic endometriotic lesions, and the regulatory effects of adiponectin on estrogen and estrogen receptor may be related to a series of pathogenetic processes, such as proliferation, adhesion, and invasion of ectopic endothelial cells.

## 6 Discussion

Most studies that have investigated the function of adiponectin have primarily been based on rodents and cellular modeling. Many of the effects of adiponectin appear to be paradoxicales with adiponectin, such as its proven ability to increase systemic insulin sensitivity, however, insulin resistance can also occur at high adiponectin levels (Kalkman, 2021). In some highly inflammatory diseases, serum adiponectin levels are high and are positively correlated with disease progression, while low adiponectin levels have been observed in some diseases in the absence of pronounced inflammation, with the expression levels being negatively correlated with disease severity (Brezovec et al., 2021). Furthermore, low levels of adiponectin have been shown to be associated with an increased prevalence of diseases such as cardiovascular disease and type 2 diabetes (Fantuzzi, 2008; Achari and Jain, 2017; Choi et al., 2020). However, in some cases, high levels of adiponectin may not be associated with any beneficial effects and may even be harmful (Francischetti et al., 2020). The paradoxes and controversies surrounding the actions of adiponectin must be further investigated.

There is evidence suggesting that low levels of adiponectin may be present in EMs and that they are associated with disease severity, a possibility that is supported by the findings of a recent meta-analysis. Therefore, adiponectin may be a beneficial factor that protects against EMs. However, there are many questions still to answer, including whether there is a clear, specific reduction in the expression level of adiponectin in EMs, whether its expression levels differ at various sites such as in ectopic lesions, PF, and serum, and whether there these levels correlate with disease severity. In addition, EMs tends to be accompanied by a low BMI, which has been reported to be negatively correlated with adiponectin levels. However, this does not explain the low BMI and smaller than expected concentrations of adiponectin in many patients with EMs, as suggested by the current research evidence. These paradoxical observations could be related to the fact that the release of adiponectin depends on the quality rather than the quantity of adipose tissue, although this must be investigated further. Additionally, it will be necessary to determine whether there is an optimal window in which the adiponectin concentration protects against EMs, and it is currently unclear whether adiponectin could serve as a clinical and/or biochemical marker of EMs.

**FIGURE 3:**
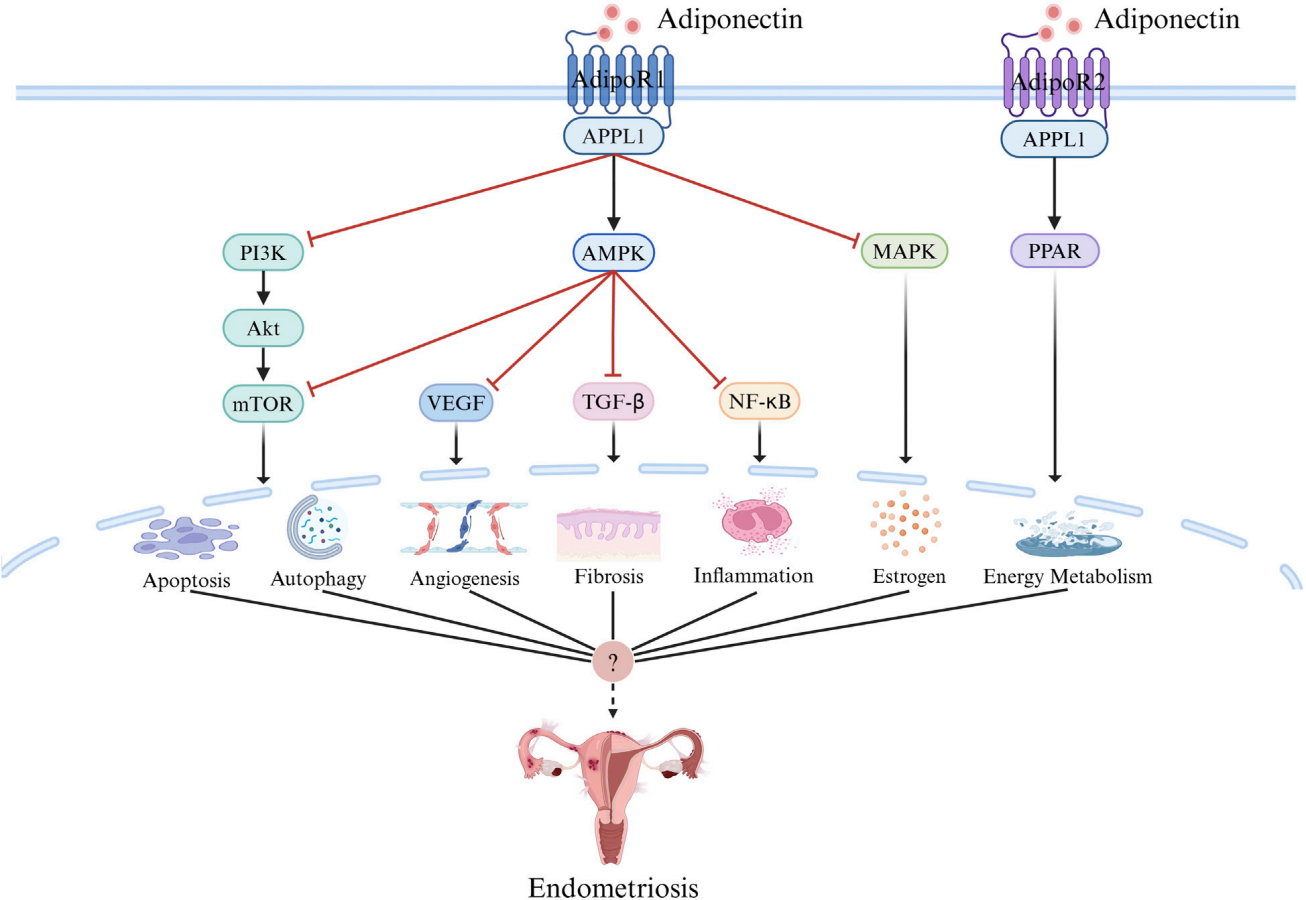
Adiponectin Signaling. Adiponectin signal transduction mainly depends on receptor ligand interaction, in which adiponectin binds to AdipoR1 and AdipoR2, and through AMPK,PPAR, PI3K/AKT, MTOR, MAPK, NF-κB, and other pathways initiate the activation of intracellular signal cascades and play a role in apoptosis, autophagy, inflammation, angiogenesis, fibrosis, energy metabolism, estrogen and other aspects, which may be related to the pathogenesis of endometriosis. Created with BioRender.com.

Relatively few studies have investigated the role of adiponectin in the pathogenesis of EMs at the clinical and cellular levels, although most have confirmed that adiponectin is an important participant in many biological processes, including apoptosis, autophagy, angiogenesis, inflammatory responses, fibrosis, energy metabolism, and estrogen-mediated effects, and an important correlation has been shown between many factors and their related pathways and the pathogenesis of EMs (Figure 3). However, many unexpected and interesting dual biological functions of adiponectin have been identified, including the bidirectional regulation of apoptosis, inflammation, and angiogenesis, in which there are still many questions that need to be answered. There is currently insufficient data to definitively conclude whether adiponectin plays a protective role against EMs. The relationship between adiponectin and EMs has many mysteries to be explored, and elucidating its role in the pathogenesis of EMs may provide important insights into the pathogenesis and treatment of EMs.

## Figures and Tables

**Table:** 

**AdipoR1**	adiponectin receptor 1
**AdipoR2**	adiponectin receptor 2
**Akt**	protein kinase B
**AMPK**	adenosine monophosphate-activated protein kinase
**α-SMA**	alpha-smooth muscle actin
**ATG13**	autophagy-related 13
**Bcl-2**	B-cell lymphoma 2
**BMI**	body mass index
**Cx43**	connexin 43
**CXCL1**	CXC motif chemokine ligand 1
**CYP2R1**	cytochrome P450 2R1
**ELT3**	Eker leiomyoma tumor-3
**EMs**	endometriosis
**eNOS**	endothelial nitric oxide synthase
**ERK**	extracellular signal-regulated kinase
**E2**	estradiol
**FABP4**	fatty acid binding protein 4
**FIP200**	FAK family kinase-interacting protein of 200 kDa
**GBP-28**	gelatin-binding protein 28
**HMEC-1**	human microvascular endothelial cell 1
**HMW**	high-molecular weight
**HUVECs**	human umbilical vein endothelial cells
**IGFBP1**	insulin-like growth factor binding protein 1
**IL-1β**	interleukin 1 beta
**IL-6**	interleukin 6
**IL-8**	interleukin 8
**IL-10**	interleukin 10
**LC3**	microtubule-associated protein light chain 3
**LKB1**	liver kinase B1
**LMW**	low-molecular weight
**LPS**	lipopolysaccharide
**MAPK**	mitogen-activated protein kinase
**MCP-1**	monocyte chemoattractant protein 1
**MMD2**	monocyte to macrophage differentiation associated 2
**MMPs**	matrix metalloproteinases
**MMW**	medium-molecular weight
**MRC-1**	mannose receptor C-Type I
**mTOR**	mammalian target or rapamycin
**NF-κB**	nuclear factor kappa B
**OA**	osteoarthritis
**PCOS**	polycystic ovary syndrome
**PF**	peritoneal fluid
**PI3K**	phosphoinositide 3-kinase
**PPAR-α**	peroxisome proliferator-activated receptor alpha
**PPAR-γ**	peroxisome proliferator-activated receptor gamma
**RA**	rheumatoid arthritis
**ROCK2**	Rho-associated coiled-coil containing protein kinase 2
**ROS**	reactive oxygen species
**SARDs**	systemic autoimmune rheumatic diseases
**SHBG**	sex hormone-binding globulin
**SOD**	superoxide dismutase
**STAT3**	signal transducer and activator of transcription 3
**STK11**	serine/threonine kinase 11
**T-cad**	T-cadherin
**TGF-β**	transforming growth factor beta
**TNF-α**	tumor necrosis factor alpha
**ULK1**	unc-51-like autophagy activating kinase 1
**VEGF**	vascular endothelial growth factor
